# Cytokines as Targets of Novel Therapies for Graves’ Ophthalmopathy

**DOI:** 10.3389/fendo.2021.654473

**Published:** 2021-04-16

**Authors:** Poupak Fallahi, Silvia Martina Ferrari, Giusy Elia, Francesca Ragusa, Sabrina Rosaria Paparo, Armando Patrizio, Stefania Camastra, Mario Miccoli, Gabriella Cavallini, Salvatore Benvenga, Alessandro Antonelli

**Affiliations:** ^1^ Department of Translational Research of New Technologies in Medicine and Surgery, University of Pisa, Pisa, Italy; ^2^ Department of Clinical and Experimental Medicine, University of Pisa, Pisa, Italy; ^3^ Section of Endocrinology, Department of Clinical and Experimental Medicine, University of Messina, Messina, Italy; ^4^ Master Program on Childhood, Adolescent and Women’s Endocrine Health, University of Messina, Messina, Italy; ^5^ Interdepartmental Program of Molecular & Clinical Endocrinology, and Women’s Endocrine Health, University Hospital, A.O.U. Policlinico Gaetano Martino, Messina, Italy; ^6^ Department of Surgical, Medical and Molecular Pathology and Critical Care, University of Pisa, Pisa, Italy

**Keywords:** corticosteroids, cytokines, Graves’ ophthalmopathy, rituximab, teprotumumab, tocilizumab

## Abstract

Graves’ disease (GD) is an organ-specific autoimmune disorder of the thyroid, which is characterized by circulating TSH-receptor (TSH-R) stimulating antibodies (TSAb), leading to hyperthyroidism. Graves’ ophthalmopathy (GO) is one of GD extra-thyroidal manifestations associated with the presence of TSAb, and insulin-like growth factor-1 receptor (IGF-1R) autoantibodies, that interact with orbital fibroblasts. Cytokines are elevated in autoimmune (i.e., IL-18, IL-6) and non-autoimmune hyperthyroidism (i.e., TNF-α, IL-8, IL-6), and this could be associated with the chronic effects of thyroid hormone increase. A prevalent Th1-immune response (not related to the hyperthyroidism *per se*, but to the autoimmune process) is reported in the immune-pathogenesis of GD and GO; Th1-chemokines (CXCL9, CXCL10, CXCL11) and the (C-X-C)R3 receptor are crucial in this process. In patients with active GO, corticosteroids, or intravenous immunoglobulins, decrease inflammation and orbital congestion, and are considered first-line therapies. The more deepened understanding of GO pathophysiology has led to different immune-modulant treatments. Cytokines, TSH-R, and IGF-1R (on the surface of B and T lymphocytes, and fibroblasts), and chemokines implicated in the autoimmune process, are possible targets of novel therapies. Drugs that target cytokines (etanercept, tocilizumab, infliximab, adalimumab) have been tested in GO, with encouraging results. The chimeric monoclonal antibody directed against CD20, RTX, reduces B lymphocytes, cytokines and the released autoantibodies. A multicenter, randomized, placebo-controlled, double-masked trial has investigated the human monoclonal blocking antibody directed against IGF-1R, teprotumumab, reporting its effectiveness in GO. In conclusion, large, controlled and randomized studies are needed to evaluate new possible targeted therapies for GO.

## Introduction

Graves’ disease (GD) is an organ-specific autoimmune disorder and it is the most frequent cause of hyperthyroidism in West Countries (1–3). It affects overall women, typically in their third to fifth decade, with an overall prevalence of 0.5% (4).

Clinical manifestations are linked to hyperthyroidism and to the autoimmune process. GD-associated signs and symptoms can vary markedly, influencing the overall well-being (5, 6).

GD is one of the principal autoimmune thyroid disorders (AITD), which are associated with the failure of immune tolerance against thyroid antigens [thyroid-stimulating hormone (TSH) receptor (TSH-R), thyroid peroxidase (TPO), and thyroglobulin (Tg)] (7, 8). At the basis of GD, an autoimmune multifactorial mechanism is present, which acts through environmental and endogenous factors in genetically predisposed individuals (9). GD is characterized by thyrotoxicosis, circulating anti-thyroid antibodies (ATA) and autoreactive lymphocytes into the thyroid (10).

The autoimmune reaction in GD induces the release of anti-TSH-R autoantibodies (TRAb) by B-cell clones, which infiltrate the thyroid, and are implicated in GD pathogenesis and its extrathyroidal manifestations, such as pretibial myxedema (PTM)/Graves’ dermopathy and Graves’ ophthalmopathy (GO). TRAb can be distinguished in: neutral antibodies; thyroid blocking antibodies (TBAb); thyroid stimulating antibodies (TSAb) (11). TSAb have similar downstream effects such as the binding of TSH to TSH-R, leading to thyrocytes proliferation, and secretion of thyroid hormones (TH; T4 and T3) (11, 12).

GD is associated to another autoimmune disease in ~20% of patients (13). A study evaluated prospectively the prevalence of other autoimmune diseases in GD patients (including some GO patients) vs. healthy controls, or patients with autoimmune thyroiditis (AT), or with multinodular goiter (MNG) (gender- and age-matched, and with a similar iodine intake). In 1.5% of GD, three associated autoimmune disorders were present. Patients with GO had a higher prevalence (18.9%) of another autoimmune disorder than GD patients without GO (15.6%) (13).

## Graves’ Ophthalmopathy

According to the European Group on Graves’ Orbitopathy (EUGOGO), GO has a prevalence of 10/10,000 persons and 16/10,000 persons in Europe and Japan, respectively (14, 15).

Approximately 30% to 50% of patients with GD develop GO. Tearing, proptosis, periorbital edema, and diplopia are the characteristic signs of Graves’ orbitopathy (16).

Orbital fibroblasts (OF) are the target of a variety of autoimmune responses, that taking together induce proliferation, adipogenesis and overproduction of the extracellular matrix, that includes glycosaminoglycans [GAG; i.e., hyaluronan (HA) and chondroitin sulfate] (4).

GD is a phasic disorder characterized during the active phase by hyperthyroidism, which can go in remission both after therapy or sometimes spontaneously. The recurrence of hyperthyroidism can occur after weeks or decades of euthyroidism, overall owing to physical or psychological stressful events (2). Also, GO is characterized by the presence of moderate-severe inflammatory signs during the active phase that can remit after therapy or spontaneously. Mild GO disappears spontaneously in the majority of cases (17). The most frequent factors associated with the recurrence of GO are stressful life events (2), and smoking (18). Smoking cessation can improve the outcome for GO (3, 4). Components of cigarette smoke might induce adipogenesis and synthesis of GAG, through reactive oxygen species (ROS) production, and whether vaping could have some negative effects on GO is still to be clarified, as nicotine induces the release of pro-​inflammatory cytokines (4, 19). As well as in GD, an autoimmune response is at the basis of GO. T cells, and autoantibodies recognizing a common autoantigen both for the thyroid and retro-ocular tissues (such as TSH-R, that is expressed also on fibroblasts and orbital preadipocytes), have a crucial role in this process (20). Moreover, another autoantigen, the insulin-like growth factor-1 (IGF-1) receptor (IGF-1R), has been suggested to be linked to GO (21). Autoantibodies against IGF-1R take part in the activation of GO OF, and its elevated expression has been shown in the thyroid and in orbital tissues in GD patients (20).

## Therapies for GO

An anti-inflammatory treatment is suggested in the active phase of GO. A euthyroid state is mandatory for successful treatment and radioiodine therapy should be avoided in active progressive GO (15).

In patients with active GO, corticosteroids (CS), or high dose intravenous (iv) immunoglobulins, decrease inflammation and orbital congestion (22). A prospective, randomized trial compared the efficacy and safety of two protocols of iv 4.5 g methylprednisolone (MP) in 80 patients, randomized to receive iv MP weekly or daily. The weekly protocol of iv MP therapy was more efficient and safer than the daily protocol for patients with active moderate-to-severe GO (23).

According to randomized controlled trials, a meta-analysis reported in patients treated with iv glucocorticoids that a reduction of 1.14 mm of proptosis and of 33% of diplopia is present, while non-randomized studies reported a reduction of 1.58 mm of proptosis and of 25% of diplopia (24). After 6 weeks, in case of worsening of the disease the therapy should be supplemented or substituted with second-line treatments (15), such as orbital radiotherapy (25, 26). Orbital decompression should be done in severe GO (27).

Patients with severe GO can have a reduced quality of life (QoL) during standard therapies and, for this reason, novel treatments, targeting directly the pathogenic disease mechanisms in GO, are necessary to improve the clinical outcome in these patients.

At present, the more deepened understanding of GO pathophysiology has led to alternative immune-modulant therapies that target various antigens (28, 29).

The aim of new treatments for thyroidal and extrathyroidal GD is firstly to target the principal autoantigens of the disease and/or molecules that have a key role in the immunological response. Future treatments of GD, and GO, will involve monoclonal antibodies (mAb) or small molecules (15).

A mechanism for targeting the pathophysiology of GD, and GO, is to “mask” the TSH-R from the action of thyroid-stimulating immunoglobulin (TSI), through the TSH-R antagonist, K1-70 (15, 30, 31). An open-label clinical trial (clinical trial number NCT02904330) is currently in progress to assess its safety and tolerability in patients with hyperthyroidism in GD (32).

A small TSH-R antagonist (clinical trial number NCGC00229600) has been shown to reduce the synthesis of HA in retro-ocular fibroblasts/adipocytes in GO, with good results (33).

Furthermore, an encouraging treatment in GO patients is teprotumumab (RV 001, R1507), a recombinant, human mAb of the immunoglobulin G1 subclass. It binds to the cysteine-rich domain of human IGF-1R with high affinity, preventing the binding with endogenous ligands (IGF-1 and IGF-2), and leading to the internalization of the receptor, in this way stopping the IGF-1R signaling cascade (34–37).

As written above, active GO is linked to the autoimmune activation of OF. This causes the production of cytokines that promote T-cell infiltration into orbital tissues, triggering a local inflammatory process, and resulting in growth and differentiation of fibroblasts, and remodeling of orbital tissues. Teprotumumab blocks these pathophysiological responses (36).

Teprotumumab decreased *in vitro* TSH-R and IGF-1R, the TSH- and IGF-1–dependent phosphorylated Akt levels in fibrocytes, such as the TSH induction of IL-6 and IL-8 mRNA and protein (38).

A randomized, double-masked study was performed, to evaluate the effectiveness and safety of this drug in 88 patients with active, moderate-to-severe GO, administered with placebo, or the drug (eight infusions) (39). The primary end point was the response in the study eye. This response was defined as a reduction of two points or more in the Clinical Activity Score (CAS) and a reduction of 2 mm or more in proptosis at week 24. About 69% of patients taking teprotumumab, in comparison to 20% of those receiving placebo, had a response at week 24 (P<0.001). At week 6, 43% of patients administered with teprotumumab and 4% with placebo had a response (P<0.001). The reported findings in active GO demonstrated that teprotumumab, compared to placebo, is more effective in decreasing proptosis and CAS (39).

Another randomized, double-masked, phase III multicenter trial was done in patients with active GO in a 1:1 ratio to receive iv teprotumumab (10 mg/kg of body weight for the 1^st^ infusion and 20 mg/kg for the following ones) or placebo once every 3 weeks for 21 weeks, for a total of 24 weeks (40). At week 24, the response in proptosis was higher in patients treated with teprotumumab than with placebo (83% vs. 10%, P<0.001). Secondary findings were significantly better with teprotumumab than with placebo (P ≤ 0.001). The data demonstrated that teprotumumab led to better outcomes (than placebo) with regard to proptosis, CAS, diplopia, and QoL, with uncommon severe adverse events (40).

Teprotumumab attained the approval by the U.S. Food and Drug Administration (FDA) as first drug for thyroid eye disease in March 2020 (41, 42).

Another emerging therapy is rituximab (RTX), a chimeric mAb against CD20. It was approved by FDA in Wegener’s granulomatosis, chronic lymphocytic leukemia, non-Hodgkin’s lymphoma, and rheumatoid arthritis (RA), and it is an off-label drug in different autoimmune diseases (43). Since RTX reduces B lymphocytes, the cytokine burden and the produced autoantibodies, its use has been proposed also in GO.

Two randomized trials showed contrasting results regarding the use of RTX. In the 1^st^ prospective, randomized, placebo-controlled trial, RTX was not effective in 25 GO patients, showing no improvement of CAS (44). In the other randomized, double-blinded study, 32 patients took iv MP or RTX, and CAS decreased in both cases, overall with RTX. After 24 weeks, all patients receiving RTX ameliorated with respect to 69% after ivMP, showing RTX was more effective than ivMP in GO patients (45).

More recently, a meta-analysis and systematic review was done in 293 GO patients, treated with RTX, or glucocorticoids, or saline, to evaluate the effectiveness of RTX. It was shown a significant decrease of CAS (vs. controls) in patients receiving RTX at 24 weeks, and a not significant one of proptosis (46).

In another study (47), 219 GO patients received pulse MP, and then oral steroids and/or orbital radiotherapy. At last, 15 (6.8%) were administered with 100 mg RTX (100–400 mg as cumulative dose) for the presence of active GO. A low dose of RTX had an anti-inflammatory effect in patients with active GO resistant to standard treatments (47). Another paper agreed with those results in 12 patients with active GO treated with a 100 mg RTX infusion; it reported that a low dose of RTX is effective in these patients, thus leading to a reduced administration of systemic steroid (48).

Furthermore, 14 patients with active and moderate-to-severe GO, of whom 11 CS-refractory, were administered with ivRTX (1000 mg twice with a 2-week interval), reporting that RTX was well-tolerated (49). A modest amelioration of CAS, and disease inactivation in half of the patients, were reported. At week 12, CAS ameliorated in 14.3% of patients and inactivation of GO in 28.6%. At week 24, total eye score and proptosis improved in 28.6% and 33% of patients, respectively.

## Cytokines and Drugs Targeting Cytokines, in GD and GO

### Cytokines in GD and GO

Cytokines [including interleukins (IL), tumor necrosis factors (TNFs), interferons (IFNs), lymphokines, and chemokines] are small proteins, important in normal physiology, and in host responses to infection, trauma, reproduction, inflammation, sepsis, and tumors (10).

They are produced by different cell types, including immune cells (B and T lymphocytes, mast cells, macrophages), fibroblasts, endothelial cells, and different stromal cells, and act through receptors (**Table 1**).

**Table 1:** Main cytokines implicated in Graves’ ophthalmopathy, the cells producing them and their biological effects.

Retrobulbar Cells	Main Cytokines	Biological effects	References
**Fibroblasts/preadipocytes**	IFN-γ, TNF-α, IL-1α has been observed in tissue sections and in primary OF cultures of patients with active GO	IFN-γ, TNF-α, IL-1α in fibroblast stimulate: -the expression of a 72-kDa heat shock protein (HSP 72) -the expression of inter-cellular adhesion molecule-1 (ICAM-I). IFN-γ, TNF-α enhance the expression of HLA-DR	(50)
	IL-6, 8, 16, RANTES, MCP-1, IFN-γ, TNF-α, IL-1	IL-16 acts as a ligand for CD4+ cells, and it is important for T-cell trafficking. IL-16 production is believed to follow that of RANTES, and both are responsible for T-cell trafficking in orbital and thyroid fibroblasts	(51)
	In retrobulbar fibroblasts and preadipocytes obtained from GO patients: 1)IFN-γ induced CXCL10 secretion in a dose-dependent manner; 2)TNF-α alone was not able to induce chemokine secretion; 3)IFN-γ+TNF-α synergistically increased CXCL10 secretion	CXCL10 induces the migration of Th1 lymphocytes into the orbit, thereby perpetuating the autoimmune cascade	(52, 53)
	In retrobulbar fibroblasts and preadipocytes obtained from GO patients: 1) IFN-γ alone dose dependently induced the secretion of CXCL9 and CXCL11; 2) IFN-γ+TNF-α combination leads to a huge response of CXCL9	C-X-C chemokines participate in the self-perpetuation of inflammation	(54)
	-Cytokines detected *in situ* in GO include TNF-α, IL-1α, IL-6, IL-8, IL-10, IL-12, IL-13, and IFN-γ; -Several of these are more highly expressed in active vs stable disease and include IL-1β, IL-6, IL-8, and IL-10; -predominance of T helper (Th)1 cytokines in active GO	-Both IFN-γ and TNF-α induce B cell activating factor in GD OF -IL-1β induces both IL-16 and RANTES in GD OF, enhancing the release of T cell migration-promoting activity	(55)
	In cultured primary OF from GO patients: -IL-17A combined with CD40L could induce the production of RANTES in time- and dose-dependent modifications; -IL-17A alone was not enough sufficient to trigger RANTES release	Amplification of GO inflammatory process	(56)
	In primary cell cultures of GO fibroblasts and preadipocytes: 1)TNF-α increases the secretion of CXCL8 dose-dependently; 2) IFN-γ stimulates the secretion of CXCL10, but it inhibits that of CXCL8	This differential modulation of CXCL10 and CXCL8 chemokines could reflect a different role of the two chemokines during the course of the disease, as CXCL10 could be associated with the initial phase of the disease when a Th1 immune response (induced by IFN-γ) is preponderant, while CXCL8 could be associated with a later chronic phase of the disease, when there is a switch to a Th2 prevalent immune response (induced by TNF-α)	(57)
**Adipocytes**	High levels of MCP-1 mRNA in the orbital fat tissue of patients with GO have been reported	MCP-1 positively correlated with the degree of macrophage infiltration in patients with GO	(58)
**Muscle cells**	In primary extraocular muscle (EOM) cultures from patients with GO: 1)IFN-γ induced CXCL10 secretion in a dose-dependent manner; 2)IFN-γ+TNF-α synergistically increased CXCL10 secretion; 3)IFN-γ and TNF-α induce CCL2 secretion	Self-perpetuation of inflammation	(59)

GO, Graves’ ophthalmopathy; IP-10, IFN-γ-inducible protein 10;C-X-C motif (CXCL)10, chemokine ligand 10; IFN-γ, interferon-γ; IL, interleukin; MCP-1/CCL2, monocyte chemoattractant protein-1; OF, orbital fibroblasts; TNF-α, tumor necrosis factor-α.

Chemokines are “chemotactic cytokines”, or signaling proteins, which can induce directed chemotaxis in the responsive cells. Chemokines exert their biological effects through the interaction with G-protein-linked transmembrane receptors, which are present on their target cells (10). The chemokine receptor (C-X-C)R3 binds Th1-chemokines [IFN-γ-inducible protein 10 (IP-10)/chemokine ligand 10 (C-X-C motif) (CXCL)10, IFN-inducible T-cell α chemoattractant (I-TAC)/CXCL11 and monokine induced by IFN-γ (MIG)/CXCL9] (60). Th1 lymphocytes are attracted in inflamed tissues by Th1 chemokines, that are released there (61), and increase cytokines production, leading to Th1 chemokines secretion by different cells, establishing an amplification feedback loop (62). Raised serum and tissue Th1 chemokines levels have been demonstrated in specific autoimmune disorders [autoimmune thyroiditis (AT) (63–65), GD and GO (52), etc.], or systemic rheumatological diseases [systemic sclerosis, psoriasis or psoriatic arthritis (66, 67), etc.], hepatitis C virus infection related autoimmune disorders (68), and cancer (69, 70).

Certain serum cytokines are elevated in non-autoimmune (IL-8, TNF-α, and IL-6) (71) and autoimmune (IL-18 and IL-6) (72) hyperthyroidism, and this could be due to the chronic effects of TH excess, and not to the coexisting autoimmune condition at the basis of GD (73).

A Th1 immune response is more prevalent in the active phase of GD, and GO; CXCR3 and Th1 chemokines (CXCL9, CXCL10, CXCL11) are crucial in this process, while a switch in immune prevalence from a Th1 to a Th2 response is present in the inactive or later phases (1, 74).

Systemic hyperthyroidism and T cell infiltration into orbital tissue leads to ROS production, which can aggravate GO severity by increasing T cell proliferation, adipogenesis, and GAG production in OF (75). Recently, the role of the protein tyrosine phosphatase 1B (PTP1B), encoded by the PTPN1 gene, has been characterized in GO (76). PTP1B is known to be involved in immune cell signaling by regulating cytokines *via* dephosphorylation of janus kinase (JAK)2, signal transducer and activator of transcription (STAT)5, and tyrosine kinase (TYK)2. After 24 h of transfection with PTPN1 siRNA, the fibroblasts were exposed to IL-1β, cigarette smoke extract (CSE), H2O2, and transforming growth factor (TGF)-β stimulations. PTPN1 silencing ameliorated ROS generation in both CSE- and H_2_O_2_-stimulated cells. The changes in the phosphorylation level of multiple transcription factors after PTP1B inhibition in GO OF indicate a more complex network of signaling pathways. PTP1B inhibition suppressed IL-1β–induced Akt, and JNK phosphorylation, but p38 MAPK phosphorylation was reduced only in GO OF. As the p38 and JNK pathways, and the MAPK pathway, can mediate the transcription and translation of inflammatory cytokines, this study demonstrated that PTP1B mediates inflammatory reactions in GO OF (76).

Inflammation and cytokine production, adipogenesis, and HA synthesis are the prevailing processes implicated in the pathogenesis of GO (20). In the initial phases of GO, the prevalent Th1 immune response facilitates cell-mediated immunity, leading to the production of IFN-γ, TNF-α, IL-1β, and IL-2, that increase fibroblast proliferation and GAG synthesis. IFN-γ induces the secretion of Th1 chemokines by fibroblasts, and the migration of lymphocytes is promoted (54). IL-1β further stimulates the synthesis of GAG (77) and the production of CCL2, CCL5, IL-6, IL-16, IL-8, and neutrophils, by OF (78). Then, inflammation leads to Th2 lymphocytes activation, that release cytokines (i.e., IL-13, IL-10, IL-5, and IL-4), and to humoral reactions and the production of IgG (79). Tissue remodeling and fibrosis characterize the late phase of GO (20).

OF express also the costimulatory protein CD40, and its binding to the ligand CD154, on T cells, induces the production of different inflammatory mediators by OF [CCL2, IL-1α, prostaglandin E2 (PGE2), IL-6, and IL-8] (80–82).

GD OF include cell subsets with specific cellular markers, such as the CD34+ CXCR4 + Collagen 1+ phenotype (CD34+ OF), while CD34− OF do not display them. On the other hand, OF obtained from normal subjects are CD34− OF (55). The axon guidance glycoprotein Slit2 is produced by CD34− OF, and it inhibits fibrocyte differentiation and modulates their characteristic gene expression profile. Slit2 upregulates IL-12 expression and it attenuates that of IL-23, which are both involved in GD and GO (83). CD34+ fibrocytes obtained from peripheral blood mononuclear cells (PBMCs) produce low basal levels of HA, very few of which were affected by bovine thyrotropin (bTSH). On the other hand, GD OF synthesize higher levels of HA, both basally and after the treatment with bTSH. The treatment of confluent cultures with rhSlit2 increases HA production in fibrocytes, while the knock-down of Slit2 expression attenuates its synthesis in GD OF. Considering HA synthase isoenzymes (HAS1–3), low HAS1 levels are present in fibrocytes, while HAS2 is the most strongly expressed in GD OF. Slit2 alters the pattern of HAS and uridine diphosphate (UDP) glucose dehydrogenase (UDPGD) expression and cytokine production, both in GD OF and fibrocytes. The exogenous rhSlit2 causes a transition from HAS1 dominating expression to that of HAS2 in fibrocytes, decreasing also the expression of TNF-α and IL-6. These data suggest that HA synthesis in GD OF depends on the CD34− OF cell subset, while TNF-α and IL-6 expression is present in CD34+ OF. For these reasons GD OF, comprising both CD34+ OF and CD34− OF, can generate inflammatory factors (84, 85).

The activation of OF by TRAb suggests the link between GD and GO. The increased OF activity contributes to the fibrosis of the orbital tissues, causing inflammatory cell infiltration, and edema. Consequently, the optic nerve can be compressed leading to optic neuropathy. The inflammation and swelling of the eye muscles are involved in the final exophthalmos (20).

Furthermore, TSH-R–expressing T cells, which could be activated by TRAb, could stimulate adipogenesis of OF in GO through a peroxisome proliferator-activated receptor-γ (PPAR-γ) ligand produced *via* upregulated cyclooxygenase (4). CD34+ fibrocytes, such as OF, migrate from the circulation into sites of inflammation and injury and express two of the major GO thyroid autoantigens, TSH-R and thyroglobulin (4). Both CD4+ and CD8+ T cells, and B cells, are present in the majority of GO orbits, and the level of infiltration correlates with disease activity (86). Macrophages are found in the orbits in early disease, whereas monocytes and mast cells have been associated with secretion of platelet-derived growth factor, that stimulates OF proliferation and HA production, in particular the platelet-derived growth factor-BB isoform in OF from both patients with/without GO (4). Mast cells also produce prostaglandins, which are able to enhance adipogenesis (87). In light of the above, there are multiple overlapping factors in the development of GO, and cytokines are strongly involved in its pathogenesis.

## Drugs Targeting Cytokines, in GD and GO

Drugs that target cytokines (etanercept, tocilizumab, infliximab, adalimumab) have been tested in GO, with encouraging results.

OF have an increased expression of TSH-R, and a strong up-regulation of TNF-α and IL-6, in the pathogenesis of GO (88).

The association between elevated circulating TNF-α levels and the severity of GO has led to the use of mAb against TNF-α, such as infliximab and etanercept (89, 90). The effectiveness of infliximab in severe steroid and surgical-resistant GO has recently been demonstrated in three cases, with complete resolution after three doses of 5 mg/kg body weight given 1 month apart (91, 92). Moreover, a positive effect of infliximab administration on active GO in a 58-year-old woman with GD has been described, in whom a single dose of this drug resulted in a reduction of inflammation and improvement of visual function, determined by magnetic resonance imaging and CAS and NO SPECS scales, with no noticeable short-term side effects (93).

Etanercept is usually used to treat RA (29). The use of etanercept has been evaluated as potential treatment in 10 patients with active GO, showing remission in 6/10 (89). Moreover, a paper reported the case of a woman, initially diagnosed with primary hypothyroidism (in substitutive treatment with levothyroxine) and subsequently with RA, who had insufficient therapeutic effectiveness with a conventional medication. After 3 years, she showed symptoms and signs of GO. Then, the patient received etanercept for RA, and after 4 months, the ocular symptoms ameliorated and exophthalmos decreased, showing that RA and GO can share similar pathogenic characteristics (88).

Furthermore adalimumab, that has been approved by FDA for psoriatic arthritis, ankylosing spondylitis, inflammatory bowel disease, RA, showed a significant amelioration in the inflammatory composite score in a retrospective review (94).

In patients with active GO, IL-6 and its soluble receptor are activated. Tocilizumab is a humanized mAb recognizing the IL-6R, which attained the approval in Castleman’s disease, systemic juvenile idiopathic arthritis, and RA. A prospective non-randomized study has been conducted in 18 GO patients (refractory to CS) treated with tocilizumab (95). An amelioration of proptosis was reported in 13 patients, extraocular motility in 15, and 7/13 solved the problem of diplopia, with no relapse of GO at the end of the follow-up. These data suggested that tocilizumab might be effective in GO patients resistant to steroids (95). Furthermore, a reduction in extraocular muscle thickness and chemosis was reported after therapy with tocilizumab in patients with GO, by using an optical coherence tomography. Four women and one man with a median age of 52 years (range, 38–73 years) were enrolled. Median GO activity duration was 17 months (12–18). After tocilizumab, median muscle thicknesses and chemosis reduced. Median CAS decreased from 5 (4–8) to 1 (0–3) (96). More recently, a paper reported three GO patients refractory to CS or with advanced diplopia, receiving tocilizumab (8 mg/kg; monthly iv), and it showed a significant amelioration in eye symptoms (97).

## Conclusion

GO is one of the extrathyroidal manifestations of GD. In patients with GO, OF can differentiate into myofibroblasts or adipocytes, able to interact with mononuclear cells, that produce chemoattractants and cytokines, in this way reiterating orbital inflammation.

Certain cytokines are elevated in autoimmune (i.e. IL-18 and IL-6) and non-autoimmune hyperthyroidism (i.e. TNF-α, IL-8, and IL-6), supporting the idea that this may be associated with the chronic effects of TH increase, and not with the GD inflammatory, autoimmune condition.

A prevalent Th1 immune response is reported in the immune-pathogenesis of GD and GO; Th1 chemokines (CXCL9, CXCL10, CXCL11), and the CXCR3, are crucial in this process

Patients with active, mild GO usually benefit from local therapies and selenium, whereas patients with moderate-to-severe disease generally need the addition of iv glucocorticoids. In case of an inadequate response to glucocorticoid therapy, different second-line therapies have been evaluated, such as orbital radiotherapy (with additional glucocorticoids), RTX, mycophenolate mofetil, cyclosporine, and methotrexate. Novel biologic agents, in particular teprotumumab and tocilizumab, have shown strong reductions in disease activity and severity. If these data are confirmed in the future, the treatment paradigm could be changed. Moreover, new immunotherapies are now evaluated for GD, that may have treatment implications also for GO (30).

At present, the more deepened understanding of GO pathophysiology has led to different immune-modulant treatments. Cytokines, TSH-R and IGF-1R (on the surface of B and T lymphocytes, and fibroblasts), and chemokines implicated in the autoimmune process, are possible targets of novel therapies.

The remodeled tissues in GO are dominated by adipogenesis, the increase of HA into the orbit, and the local synthesis of proinflammatory cytokines (including TNF-α and IL-6). There is a high complexity in the interactions among the cells of the heterogeneous population of GD OF in the GO orbit. Slit2 seems to play an important role in the determination of the pattern of HAS and UDPGD expression and IL-6, TNF-α, and HA production in these fibroblasts, and it could be considered as an interesting therapeutic target in GO (84, 85).

In the pathogenesis of GO, OF have an increased expression of TSH-R, and a strong up-regulation of TNF-α and IL-6. Drugs that target cytokines have been tested in GO, with encouraging results. The association between elevated circulating TNF-α levels and the severity of GO has led to the use of mAb against TNF-α, such as infliximab, adalimumab, and etanercept. Also tocilizumab (anti-IL6-R) have reached significant findings in GO.

In conclusion, large, controlled and randomized studies are needed to evaluate new possible targeted therapies for GO.

## Author Contributions

PF, SMF, SB, and AA conceived the paper. All authors contributed to the article and approved the submitted version.

## Conflict of Interest

The authors declare that the research was conducted in the absence of any commercial or financial relationships that could be construed as a potential conflict of interest.
